# TGF-β Signaling as a Pathological Continuum Linking Idiopathic Pulmonary Fibrosis and Lung Cancer

**DOI:** 10.3390/cells15050480

**Published:** 2026-03-06

**Authors:** Kuo-Liang Huang, Lu-Kai Wang, Fu-Ming Tsai

**Affiliations:** 1 Division of Pulmonary Medicine, Taipei Tzu Chi Hospital, Buddhist Tzu Chi Medical Foundation, New Taipei City 231, Taiwan; klhuang66@gmail.com; 2 School of Medicine, Tzu Chi University, Hualien 970, Taiwan; 3 National Center for Biomodels, National Institutes of Applied Research, Taipei 115, Taiwan; 2407026@niar.org.tw; 4 Department of Research, Taipei Tzu Chi Hospital, Buddhist Tzu Chi Medical Foundation, New Taipei City 231, Taiwan

**Keywords:** TGF-β signaling, idiopathic pulmonary fibrosis, lung cancer, epithelial–mesenchymal transition, canonical and non-canonical pathways, fibroblast activation, tumor microenvironment

## Abstract

**Highlights:**

**What are the main findings?**

- Persistent and dysregulated TGF-β signaling constitutes a shared pathogenic axis linking idiopathic pulmonary fibrosis and lung cancer through coordinated effects on epithelial cells, fibroblasts, and the immune microenvironment.
- The temporal intensity and cellular context of TGF-β activation critically determine its transition from physiological repair to pathological fibrosis, immune suppression, and tumor progression.

**What are the implications of the main findings?**

- Therapeutic targeting of TGF-β signaling requires precision strategies that distinguish pathological activation from its essential physiological functions to avoid adverse effects.
- Context-specific and combinatorial approaches, particularly integrating TGF-β modulation with immunotherapy or anti-fibrotic agents, represent promising directions for future clinical translation.

**Abstract:**

Transforming growth factor-β (TGF-β) signaling plays a central role in lung tissue homeostasis, coordinating epithelial repair, immune resolution, and stromal remodeling following injury. However, persistent or dysregulated TGF-β activation is a hallmark of both idiopathic pulmonary fibrosis (IPF) and lung cancer, two devastating pulmonary diseases that are traditionally studied as distinct entities. Emerging evidence suggests that this dichotomous view may obscure shared pathogenic mechanisms driven by aberrant TGF-β signaling dynamics. In this review, we synthesize experimental, translational, and clinical findings to propose a unifying framework in which IPF and lung cancer represent endpoints along a shared TGF-β–driven pathological continuum. We highlight how the duration and intensity of TGF-β signaling determine divergent cellular outcomes across epithelial cells, fibroblasts, and immune compartments—ranging from physiological wound repair to irreversible fibrotic remodeling and the establishment of a pro-tumorigenic niche. Particular emphasis is placed on the temporal transition from acute injury responses to chronic signaling states that promote epithelial plasticity, fibroblast fixation, immune suppression, and genomic instability. By integrating fibrosis and tumorigenesis into a single pathophysiological model, this review reframes TGF-β signaling as a time-dependent disease modifier rather than a disease-specific factor. This perspective provides a conceptual basis for therapeutic strategies targeting TGF-β signaling windows to intercept disease progression before irreversible fibrosis or malignant transformation occurs.

## 1. Introduction

Pulmonary fibrosis and lung cancer are traditionally regarded as two distinct pulmonary disorders; however, accumulating evidence over the past decade has revealed a substantial overlap in their pathogenic mechanisms, microenvironmental alterations, and signaling networks. Idiopathic pulmonary fibrosis (IPF) is a progressive and irreversible interstitial lung disease characterized by repetitive alveolar epithelial injury, persistent fibroblast activation, and the excessive deposition of the extracellular matrix (ECM), ultimately leading to respiratory failure and premature mortality [1]. Epidemiological studies estimate the prevalence of IPF to range from approximately 10–60 cases per 100,000 persons worldwide, with an annual incidence of 3–9 cases per 100,000 persons. Despite therapeutic advances, the median survival remains approximately 3–5 years after diagnosis, with a five-year mortality rate comparable to or exceeding that of several major malignancies [2,3]. Notably, several hallmark pathological features of IPF closely resemble those observed in lung cancer, including epithelial–mesenchymal transition (EMT), the establishment of an immunosuppressive microenvironment, and profound alterations in cell fate determination [4].

Lung cancer remains one of the leading causes of cancer-related mortality worldwide, driven by a complex interplay of genetic susceptibility, environmental factors such as cigarette smoking, and chronic inflammatory insults. Importantly, epidemiological studies have demonstrated that patients with IPF exhibit a significantly higher incidence of lung cancer compared with the general population. Meta-analyses indicate that the cumulative incidence of lung cancer in IPF patients ranges from approximately 10–20%, with a reported 5- to 7-fold increased risk relative to age-matched controls [5,6]. These findings lend strong support to the concept that these two diseases share a convergent pathogenic axis rather than representing independent entities [4].

Among the shared molecular drivers underlying both conditions, transforming growth factor-β (TGF-β) has emerged as a central signaling hub. Under physiological conditions, TGF-β plays essential roles in tissue development, alveolar epithelial differentiation, and the maintenance of immune homeostasis. In contrast, the sustained and dysregulated activation of TGF-β signaling under pathological conditions promotes EMT, fibroblast-to-myofibroblast differentiation, and extensive ECM remodeling, thereby driving fibrotic progression while simultaneously creating a permissive microenvironment that supports tumor invasion and metastasis [4,7].

Mechanistically, in IPF, TGF-β signaling induces fibroblast differentiation into contractile myofibroblasts and upregulates ECM-associated gene expression, resulting in progressive stiffening and the architectural distortion of the lung interstitium [8]. In lung cancer, TGF-β exerts pleiotropic effects by inducing EMT in tumor cells, reshaping the immune microenvironment toward immune suppression, and promoting the formation of cancer-associated fibroblasts (CAFs), all of which collectively facilitate tumor progression and invasiveness [4].

Consistent with these functional roles, numerous studies have demonstrated that TGF-β and its receptors are markedly upregulated in both IPF and lung cancer tissues compared with normal lung tissue and that elevated TGF-β signaling correlates with disease severity and poor clinical outcomes [4]. For instance, meta-analyses have reported that high TGF-β expression in lung cancer patients is significantly associated with reduced overall survival, suggesting its potential utility as a negative prognostic biomarker [9]. Moreover, TGF-β is recognized as a potent inducer of EMT, capable of upregulating EMT-associated transcription factors such as SNAI1/2 and ZEB1/2 in non-small cell lung cancer (NSCLC) cells, thereby enhancing the migratory capacity and invasive behavior [4,10,11].

Based on this framework, the present review introduces an integrated overview model illustrating the dynamic roles of TGF-β across three pathological stages—acute tissue repair, chronic fibrosis, and pro-tumorigenic states—and subsequently elaborates on the following key themes: (i) the signaling dynamics of TGF-β in pulmonary fibrosis and lung cancer, including canonical and non-canonical pathways; (ii) the shared molecular and cellular mechanisms underlying the IPF–lung cancer pathological axis; and (iii) current and emerging therapeutic strategies targeting the TGF-β pathway and their associated challenges.

Despite decades of investigation, critical gaps remain in our understanding of how temporal dynamics, cellular context, and signaling magnitude shape divergent pathological outcomes in pulmonary fibrosis and lung cancer. Most existing reviews address oncogenic transformation or fibrotic remodeling separately, often emphasizing canonical Sma- and Mad-related proteins (SMAD) pathways without fully integrating non-canonical signaling or stage-dependent transitions [12,13,14].

**Figure 1:**
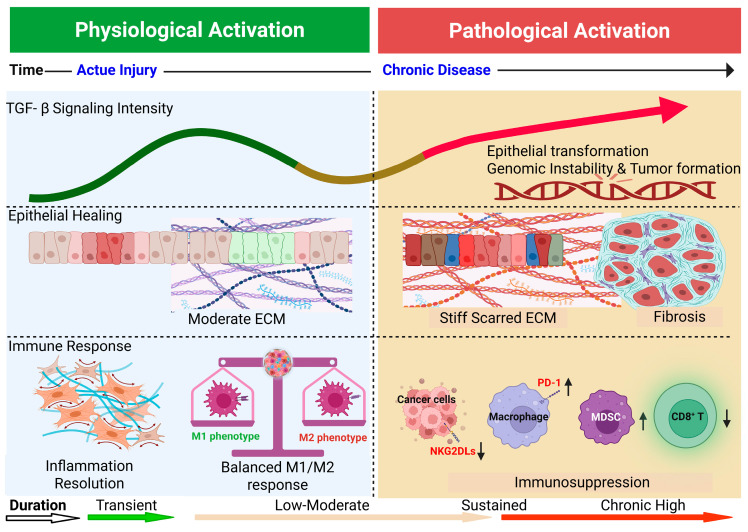
Time- and intensity-dependent effects of TGF-β signaling in lung epithelial, fibroblast, and immune compartments. This schematic illustrates a conceptual model in which the biological outcomes of TGF-β signaling are determined by its temporal dynamics and signaling intensity. The horizontal axis represents disease progression from acute lung injury to chronic lung disease, while the vertical axis denotes increasing TGF-β signaling intensity and signaling persistence (from transient/short-term to sustained/chronic activation). Epithelial compartment: Transient TGF-β activation supports epithelial repair and tissue homeostasis following acute injury. In contrast, sustained signaling promotes partial epithelial–mesenchymal transition (EMT), genomic instability, and malignant transformation. Fibroblast compartment: Short-term activation facilitates reversible wound healing, whereas prolonged TGF-β signaling drives fibroblast fixation into myofibroblasts with excessive extracellular matrix (ECM) deposition, resulting in irreversible fibrosis. Immune compartment: Early or transient signaling maintains effective immune surveillance and inflammation resolution. Persistent TGF-β exposure promotes programmed cell death protein 1 (PD-1) expression on macrophages and T cells, reduces NKG2D ligand (NKG2DL) expression on epithelial cells, and shifts macrophage polarization from an M1-like pro-inflammatory state toward an M2-like immunoregulatory phenotype, collectively reinforcing immune tolerance. Together, this integrated model highlights TGF-β as a central temporal regulator that links physiological repair to pathological fibrosis and cancer development through coordinated, cell type-specific signaling reprogramming. This figure was created in BioRender.

Here, we propose an integrated framework in which the duration and magnitude of TGF-β signaling orchestrate distinct yet overlapping trajectories across epithelial, stromal, and immune compartments, linking acute repair to chronic fibrosis and tumorigenesis (Figure 1).

## 2. Pathological Features and Molecular Regulatory Networks of Idiopathic Pulmonary Fibrosis

Pulmonary fibrosis is a chronic and progressive interstitial lung disease characterized by the excessive deposition of connective tissue within the pulmonary interstitium, leading to irreversible fibrotic remodeling that replaces the normal alveolar architecture with scar-like tissue [15]. Although pulmonary fibrosis encompasses multiple etiologies, including autoimmune, occupational, environmental, and radiation-induced causes, the present review focuses specifically on IPF as the prototypical and most extensively studied form of progressive fibrotic lung disease. IPF therefore serves as the primary mechanistic paradigm discussed throughout this section. This process results in reduced lung compliance and impaired gas exchange. IPF, the most common and severe form of pulmonary fibrosis, predominantly affects older adults and presents clinically with progressive dyspnea, persistent dry cough, and restrictive ventilatory dysfunction. Despite advances in supportive care, the median survival of patients with IPF remains only approximately 3–5 years, reflecting the irreversible nature of the disease and its limited responsiveness to current therapeutic options [1,16].

The pathogenesis of IPF involves a complex interplay between epithelial injury and dysregulated repair mechanisms. Recurrent damage to alveolar epithelial cells triggers sustained interstitial inflammation, fibroblast activation, and the accumulation of myofibroblasts, ultimately leading to excessive ECM deposition and increased tissue stiffness [17]. Morphologically, IPF is characterized by the usual interstitial pneumonia pattern, which features spatial and temporal heterogeneity, fibroblastic foci, and the presence of subpleural honeycombing cysts representing end-stage architectural destruction [18,19]. Honeycombing constitutes the defining radiological and histopathological hallmark of IPF and reflects irreversible fibrotic remodeling. Unlike normal wound healing following an acute injury, this process is driven by the failure to appropriately terminate repair signals, resulting in persistent fibrotic remodeling. As fibrosis progresses, the destruction of alveolar structures markedly reduces the effective gas exchange surface area, leading to progressive respiratory insufficiency and hypoxemia [20].

Pulmonary fibrosis is not driven by a single molecular pathway but rather arises from the coordinated action of multiple signaling cascades and cytokine networks. These include platelet-derived growth factor (PDGF), connective tissue growth factor (CTGF), fibroblast growth factors (FGFs), Wnt/β-catenin signaling, and the Hippo–YAP/TAZ pathway, as well as various inflammatory cytokines and chemokines [21,22]. Together, these pathways orchestrate aberrant repair responses following epithelial injury by promoting fibroblast activation, myofibroblast accumulation, and excessive ECM production. Among these pro-fibrotic mediators, TGF-β is widely recognized as the most central and integrative regulator. TGF-β not only directly induces fibroblast differentiation and ECM synthesis but also establishes positive feedback loops with mechanical sensing pathways and other pro-fibrotic signals, thereby sustaining a pathological fibrotic microenvironment [23]. Extensive analyses of clinical specimens and experimental models consistently demonstrate that the persistent activation of TGF-β signaling strongly correlates with disease severity and progression in pulmonary fibrosis, underscoring its pivotal role in both the disease pathogenesis and therapeutic development [24,25].

While pulmonary fibrosis arises from the convergence of multiple signaling pathways and cellular interactions, these diverse regulatory networks do not operate independently. Instead, they are integrated and coordinated by a limited number of master regulators that determine whether tissue repair resolves appropriately or progresses toward irreversible fibrosis. Among these regulators, TGF-β occupies a unique position due to its ability to simultaneously modulate epithelial plasticity, fibroblast activation, immune regulation, and extracellular matrix remodeling [15]. Therefore, to better understand how physiological repair mechanisms transition into pathological fibrosis—and how similar signaling programs are later co-opted during tumorigenesis—it is essential to examine the dual roles of TGF-β signaling under normal and disease conditions.

## 3. Roles of TGF-β in Physiological and Pathological Contexts

TGF-β represents a highly conserved family of multifunctional cytokines that play essential roles in a wide range of physiological processes, including embryonic development and organogenesis, tissue regeneration and wound healing, the maintenance of immune tolerance, and the regulation of epithelial–mesenchymal interactions [14]. In mammals, three highly homologous isoforms—TGF-β1, TGF-β2, and TGF-β3—have been identified. Although these isoforms signal through the same receptor complexes and share largely overlapping downstream SMAD-dependent and SMAD-independent pathways, their spatial expression patterns, activation thresholds, and biological effects are context-dependent [26]. TGF-β1 is the predominant isoform implicated in fibrotic lung disease; however, TGF-β3 is not exclusively anti-fibrotic and may exert milder profibrotic or regulatory effects depending on cellular context and extracellular matrix composition [27]. These nuances underscore that TGF-β signaling output reflects not only pathway activation but also isoform distribution and microenvironmental conditions.

During normal organ development and maturation, TGF-β signaling tightly controls fundamental cellular behaviors such as proliferation, differentiation, apoptosis, and migration, thereby preserving tissue homeostasis [28]. In the lung, TGF-β contributes to branching morphogenesis and alveolarization by coordinating epithelial–mesenchymal crosstalk, underscoring its indispensable role in maintaining normal pulmonary structures and functions [4].

**Figure 2:**
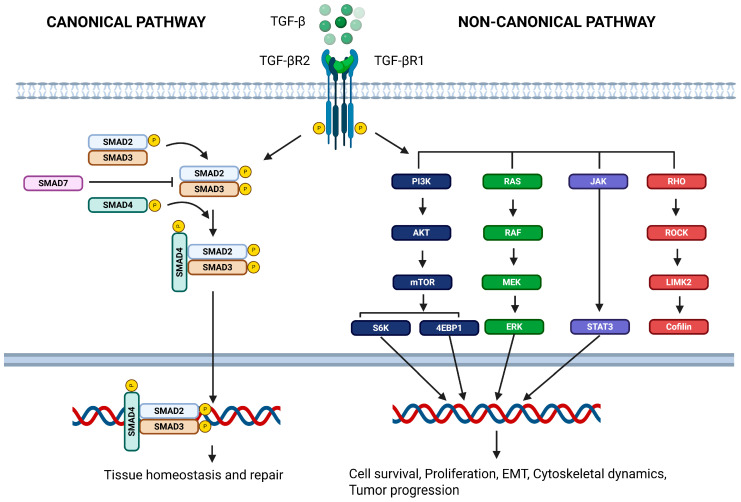
An overview of canonical and non-canonical TGF-β signaling pathways. This figure illustrates the major signaling cascades activated by transforming growth factor-β (TGF-β), including the canonical SMAD-dependent pathway and multiple non-canonical (SMAD-independent) pathways. Upon ligand binding, TGF-β receptors initiate phosphorylation and the nuclear translocation of SMAD2/3 to regulate the transcription of target genes involved in tissue homeostasis and repair. In parallel, TGF-β can activate non-canonical signaling pathways, such as PI3K/AKT, MAPK, and Rho-like GTPase signaling, which modulate cell survival, cytoskeletal dynamics, and context-dependent pathological responses. This figure was created in BioRender.

At the molecular level, TGF-β signaling is characterized by a highly regulated and context-dependent architecture. Importantly, TGF-β is not secreted in an active form. Instead, it is produced as a latent complex consisting of the mature cytokine non-covalently bound to latency-associated peptide (LAP) and further tethered to the extracellular matrix through latent TGF-β–binding proteins (LTBPs) [29]. This large latent complex sequesters TGF-β within the ECM, preventing spontaneous receptor engagement. Activation requires mechanical or proteolytic release, which constitutes a critical regulatory checkpoint in fibrotic disease. Activated TGF-β ligands initially bind to the transmembrane type II TGF-β receptor (TβRII), which subsequently recruits and phosphorylates the type I receptor (TβRI), forming an active receptor complex. In the lung—particularly in IPF—αvβ6 integrin–mediated mechanotransduction plays a central role in latent TGF-β activation. Mechanical tension generated by epithelial cells or myofibroblasts induces conformational changes in the LAP complex, releasing active TGF-β without proteolytic cleavage [30,31]. Matrix stiffening further amplifies this process, establishing a feed-forward loop in which fibrosis enhances TGF-β activation, which in turn promotes additional ECM deposition and tissue rigidity. Thus, TGF-β activation in IPF is not merely increased ligand production but is critically dependent on dysregulated mechanical activation mechanisms. This receptor activation initiates the canonical Smad pathway, whereby receptor-phosphorylated Smad2 and Smad3 associate with Smad4 and translocate into the nucleus to regulate the transcription of target genes. In parallel, TGF-β receptors can activate multiple non-canonical (non-Smad) signaling pathways, including MAPK, PI3K/AKT, and RhoA/ROCK cascades. The relative contribution of these signaling branches varies depending on the cell type, microenvironmental context, and temporal dynamics, conferring remarkable functional versatility to TGF-β signaling (Figure 2) [32].

Under physiological conditions, TGF-β signaling is subject to stringent positive and negative regulatory mechanisms. Negative feedback regulators, such as Smad7, act to restrain receptor activity and prevent excessive or prolonged signaling responses, thereby limiting unnecessary tissue remodeling [33]. TGF-β itself induces SMAD7 transcription as part of an intrinsic negative feedback loop. SMAD7 binds to activated TβRI and recruits E3 ubiquitin ligases such as Smad ubiquitination regulatory factor 1 (SMURF1) and SMURF2, promoting receptor ubiquitination and degradation, thereby attenuating downstream signaling [34]. However, in pathological contexts including IPF and lung cancer, this regulatory circuit appears impaired. Mechanistic studies suggest that enhanced SMURF-mediated degradation of inhibitory components, epigenetic silencing of SMAD7, microRNA-mediated repression (e.g., miR-21), and reduced protein stability collectively weaken negative feedback control [35,36,37]. The disruption of this inhibitory checkpoint permits sustained SMAD2/3 activation and contributes to persistent fibrotic and tumor-promoting signaling. However, under conditions of chronic injury or persistent stimulation, this regulatory balance becomes disrupted. Sustained TGF-β activation then emerges as a central driver of pathological fibrosis. Aberrantly elevated TGF-β signaling promotes fibroblast differentiation into myofibroblasts, enhances ECM production and cross-linking, and suppresses ECM degradation. Concurrently, TGF-β induces epithelial plasticity through a partial or complete EMT, while impairing effective epithelial regeneration, ultimately leading to an irreversible fibrotic architecture [4,12]. However, the precise contribution of EMT to the myofibroblast pool in IPF remains a matter of ongoing debate. Early in vitro and animal studies suggested that epithelial cells could undergo EMT and directly transition into collagen-producing fibroblasts. In contrast, lineage-tracing studies in genetically engineered mouse models have demonstrated that, although epithelial cells exhibit partial EMT phenotypes and contribute to profibrotic signaling, they rarely complete full transdifferentiation into functional myofibroblasts in vivo [38,39]. These findings suggest that EMT in IPF may primarily represent a state of epithelial plasticity that amplifies fibrogenic signaling rather than serving as a dominant cellular source of myofibroblasts.

In pulmonary fibrosis—particularly IPF—substantial evidence supports the notion that the persistent activation of TGF-β signaling is a key determinant of disease progression [40]. TGF-β is abundantly produced by injured epithelial cells, activated macrophages, and other stromal components within the fibrotic lung, establishing local positive feedback loops that further amplify its signaling output [20]. This sustained activation drives fibroblast–myofibroblast conversion and excessive ECM deposition, while simultaneously interacting with other pro-fibrotic pathways, including Wnt/β-catenin, PDGF, and CTGF signaling. Moreover, TGF-β intersects with immune regulatory circuits, further reinforcing a chronic, non-resolving fibrotic microenvironment. Collectively, this transition from tightly regulated physiological repair to uncontrolled pathological signaling represents a shared mechanistic foundation across multiple organ fibroses, including those affecting the lung, liver, and kidney. Consequently, therapeutic strategies targeting the TGF-β/Smad axis have become a central focus in the development of anti-fibrotic interventions [12,22].

## 4. Pulmonary Carcinogenesis: Etiology and the Impact of Fibrotic Lung Disease

Lung cancer remains the leading cause of cancer-related mortality worldwide and is characterized by marked pathological and clinical heterogeneity. Histologically, lung cancer is broadly classified into NSCLC and small cell lung cancer (SCLC), with NSCLC accounting for approximately 80–85% of all cases, among which lung adenocarcinoma is the most prevalent subtype [41]. Although advances in low-dose computed tomography screening and molecular targeted therapies have improved outcomes in selected patient populations, the overall five-year survival rate of lung cancer remains poor, underscoring the need for a deeper understanding of its underlying mechanisms and early disease evolution [42].

The development of lung cancer is the result of the long-term accumulation of multiple risk factors. Cigarette smoking remains the most well-established and dominant carcinogenic factor; however, a substantial proportion of lung cancer cases occur in never-smokers, highlighting the importance of additional etiological contributors. Environmental and occupational exposures (such as fine particulate matter PM2.5, radon, and asbestos), inherited and somatic genetic alterations (including mutations in Epidermal Growth Factor Receptor (*EGFR*), Kirsten Rat Sarcoma Viral Oncogene Homolog (*KRAS*), and Tumor Protein 53 (*TP53*), and chronic inflammation and tissue injury have all been shown to significantly increase lung cancer risk [43]. At the molecular level, oncogenic driver mutations and dysregulated intracellular signaling promote uncontrolled proliferation, resistance to apoptosis, and genomic instability, thereby forming the foundation of lung carcinogenesis [44,45].

In recent years, chronic lung diseases—particularly IPF—have increasingly been recognized as independent risk factors for lung cancer development [6]. Epidemiological studies consistently demonstrate that patients with IPF exhibit a markedly elevated risk of lung cancer compared with the general population, with cumulative incidence rates rising with disease duration and reaching approximately 10–15% within five years of diagnosis [46,47,48]. Notably, although not all lung cancers arise directly from fibrotic lung disease, tumors in IPF patients frequently develop within fibrotic or scarred regions of the lung and often display distinct histological and molecular characteristics compared with lung cancers arising in non-fibrotic lungs.

From a mechanistic perspective, pulmonary fibrosis and lung cancer share several key biological features, including chronic epithelial injury, dysregulated repair responses, ECM remodeling, immune microenvironment imbalance, and the persistent activation of pro-fibrotic and pro-tumorigenic signaling pathways [49]. These alterations not only drive aberrant fibroblast activation and tissue stiffening but may also impose selective pressure on epithelial cells, thereby facilitating the accumulation of genetic alterations and malignant transformation [49]. Consequently, lung cancer arising in the context of chronic lung injury and fibrosis is increasingly viewed as a disease driven by abnormal repair processes, rather than solely by isolated carcinogenic insults.

Overall, lung carcinogenesis reflects the complex interplay between environmental exposures, genetic susceptibility, and chronic pathological conditions. The pro-fibrotic and pro-tumorigenic microenvironment established by pulmonary fibrosis provides a critical disease continuum—the so-called fibrosis–cancer continuum—that offers an integrative framework for understanding lung cancer development and sets the stage for subsequent discussions on key signaling pathways, including TGF-β, that link fibrosis and cancer progression [50].

## 5. Genetic Alterations in the TGF-β Signaling Pathway and Their Roles in Pulmonary Fibrosis and Lung Cancer

Under physiological conditions, TGF-β activation is tightly regulated to facilitate tissue repair and immune tolerance; however, the chronic or excessive activation of this pathway may lead to pathological consequences, including pulmonary fibrosis and lung cancer. To comprehensively understand genetic-level alterations in TGF-β signaling in pulmonary fibrosis and lung cancer, this section summarizes findings from previously published large-scale genomic analyses based on public databases, followed by evidence from candidate gene studies focusing on single nucleotide polymorphisms (SNPs), somatic mutations, and epigenetic modifications. No independent data mining or primary interrogation of The Cancer Genome Atlas (TCGA) or genome-wide association studies (GWAS) databases was performed in this review; all genomic-scale information presented below is derived from previously published studies.

### 5.1. Genomic-Scale Analyses of TGF-β Pathway Genes in Lung Cancer and IPF

As summarized in Table 1, genome-wide analyses of lung cancer datasets—particularly those from TCGA—as reported in previously published studies indicate that core components of the TGF-β signaling pathway such as *TGFB1*, *TGFBR1*, *TGFBR2*, and *SMAD2/3*—are not among the high-frequency driver genes in lung cancer. This contrasts sharply with canonical oncogenic drivers in lung cancer, including EGFR, KRAS, and TP53. Although alterations in TGF-β pathway activity are frequently observed in lung tumors, these changes are rarely attributable to recurrent coding mutations. Instead, they more commonly reflect downstream signaling dysregulation, transcriptional imbalance, or epigenetic modulation rather than the direct genomic disruption of pathway genes. Importantly, all genomic-scale information summarized here is derived from previously published TCGA and GWAS, rather than independent database analyses conducted in the present review.

Among TGF-β-related genes, *SMAD4* represents one of the few examples in which somatic alterations have been detected in a subset of NSCLC cases. Loss-of-function mutations or chromosomal deletions of *SMAD4* have been associated with tumor progression and poor prognoses. Nevertheless, the overall frequency of *SMAD4* alterations remains relatively low, reinforcing the notion that TGF-β pathway genes are not typical mutation-driven oncogenic initiators in lung cancer. Notably, several studies have suggested that epigenetic regulation may significantly influence TGF-β signaling activity even in the absence of direct genetic mutations.

In contrast, genome-wide association studies (GWAS) of IPF reveal a distinct pattern of genetic susceptibility. To date, core TGF-β pathway genes (e.g., *TGFB1*, *SMAD2*, *SMAD3*, and *SMAD4*) have not emerged as significant GWAS loci. Instead, the strongest associations are consistently observed in non-*TGF-β* genes. A prominent example is the MUC5B promoter polymorphism rs35705950, in which the T allele confers a several-fold increased risk of IPF and has been reproducibly validated across European and Asian populations [54]. Additionally, variants in telomere maintenance genes, such as telomerase reverse transcriptase and telomerase RNA component, have been identified as IPF risk factors, further supporting the concept that IPF susceptibility is primarily driven by genetic determinants of epithelial integrity, telomere stability, and mucociliary clearance rather than direct mutations in TGF-β signaling genes [55].

### 5.2. Gene-Level Variants and the Epigenetic Regulation of the TGF-β Pathway

Beyond common GWAS markers, candidate gene studies have demonstrated that polymorphisms in TGF-β pathway genes may be associated with lung cancer susceptibility or clinical heterogeneity (Table 2). For example, the *TGFB1* promoter polymorphism −509C/T (rs1800469), along with other SNPs such as rs1982073, has been demonstrated to correlate with lung cancer risk in multiple population-based studies [56]. In a Korean case–control cohort, carriers of the −509T allele exhibited a reduced risk for certain lung cancer subtypes, suggesting that these variants may influence TGF-β1 expression levels and functional output, thereby modulating individual susceptibility [57]. Similarly, polymorphisms in *TGFB1* and *TGFBR2* have been investigated in non-smoking female lung adenocarcinoma. Evidence suggests that the *TGFB1* C509T variant may interact with environmental exposures, such as cooking oil fumes, resulting in a modified lung cancer risk in specific subpopulations [58].

In addition to SNPs, epigenetic alterations affecting TGF-β signaling components have gained increasing attention. The promoter hypermethylation of SMAD4 has been observed in lung cancer tissues arising in IPF patients, leading to reduced SMAD4 expression and impaired growth inhibitory responses to TGF-β [60]. Moreover, the hypermethylation of *SMAD7*, an inhibitory regulator of TGF-β signaling, has been linked to enhanced metastatic potential in lung adenocarcinoma through pathway hyperactivation mediated by DNMT3B recruitment [61].

Despite these observations, the effects of gene-level variants in the TGF-β pathway are generally modest and population-dependent and fail to achieve genome-wide significance in large GWAS. This suggests that such variants function primarily as susceptibility modifiers rather than direct oncogenic drivers, a conclusion consistent with findings from IPF GWAS that do not implicate TGF-β core genes as major independent risk loci.

Importantly, TGF-β signaling functions as an integrative regulatory hub, and its pathological activation is closely linked to the comorbidity of pulmonary fibrosis and lung cancer. Multiple reviews have demonstrated markedly elevated TGF-β signaling activity in IPF lung tissue, where sustained activation promotes myofibroblast differentiation, ECM deposition, and the establishment of a pro-fibrotic and immunosuppressive microenvironment [20,62]. Such conditions may facilitate preneoplastic transformation and tumor development [2]. Similarly, in NSCLC, TGF-β signaling has been proven to induce EMT, enhance invasive and metastatic capacities, and correlate with adverse clinical outcomes [4].

In summary, integrated evidence from the TCGA and IPF GWASs indicates that direct mutations in core TGF-β pathway genes are not common genetic drivers of pulmonary fibrosis or lung cancer. Nevertheless, genetic and epigenetic variations within this pathway may modulate individual responsiveness to pathological TGF-β activation. More critically, sustained functional dysregulation and the chronic activation of TGF-β signaling—particularly within the fibrotic lung microenvironment—are tightly linked to disease progression, underscoring the pathway’s significance as both a therapeutic target and a prognostic indicator.

## 6. The Functional Activation of the TGF-β Signaling Pathway in Pulmonary Fibrosis and Lung Cancer

The preceding sections have described how genetic mutations or polymorphisms in TGF-β pathway-related genes may influence the risk of pulmonary fibrosis and lung cancer. However, accumulating evidence indicates that even in the absence of overt genetic alterations in *TGFB1*, *TGFBR1/2*, or *SMAD* family genes, TGF-β signaling can remain persistently activated through excessive ligand production, enhanced receptor activation, or the amplification of downstream signaling cascades, thereby driving pathological progression [63,64]. Accordingly, this section focuses on the impact of the functional activation of the TGF-β pathway in a genetically intact background on the development of pulmonary fibrosis and lung cancer (Table 3).

### 6.1. TGF-β Pathway Activation in Pulmonary Fibrosis

In lung tissues from patients with IPF, TGF-β1 expression is markedly elevated and is accompanied by sustained phosphorylation and the nuclear translocation of TGF-β receptors (TGFBR1/2) and downstream SMAD2/3, indicating a chronic and insufficiently terminated activation state of this signaling axis [69]. Importantly, most studies have not identified structural mutations in TGFB1 or TGFBR genes, suggesting that aberrant TGF-β signaling in IPF predominantly arises from amplification and dysregulation at the signaling level, rather than from genomic alterations.

In bleomycin (BLM)–induced experimental models of pulmonary fibrosis, the TGF-β–SMAD3 axis has been firmly established as a central regulator of fibroblast-to-myofibroblast differentiation and the excessive deposition of collagen and other ECM components [70]. Functional studies further demonstrate that pharmacological inhibition or signaling-level interference that effectively reduces SMAD3 phosphorylation and transcriptional activity significantly attenuates the fibrotic severity—even in the absence of any genetic modification or knockout of TGFB1 or SMAD3 [66,71].

Recent studies further indicate that TGF-β-induced pulmonary fibrosis is not mediated solely by the canonical SMAD2/3 pathway [12,72]. In the pathological milieu of IPF, TGF-β simultaneously activates multiple non-canonical (non-SMAD) signaling pathways, including PI3K/AKT, p38 MAPK, and JNK, which engage in extensive crosstalk with canonical Smad signaling to amplify fibrotic responses. For example, in primary human lung fibroblasts, TGF-β1 drives profibrotic gene expression through coordinated activation of the PI3K, JNK, and Akt signaling pathways, and pharmacological inhibition of these kinases markedly reduces TGF-β1-induced responses [73]. Furthermore, TGF-β1 induces EMT in alveolar epithelial cells partially through p38 MAPK and JNK activation, where blockade of these kinases attenuates EMT phenotypes, illustrating functional roles for non-SMAD signaling in epithelial plasticity linked to fibrosis and tumorigenesis [74]. Additionally, TGF-β1 activates the PI3K-Akt-mTORC1 axis to reprogram lung fibroblast metabolism and drive collagen synthesis, and PI3K inhibition prevents downstream profibrotic signaling, underscoring the significance of sustained non-canonical pathway engagement [75].

**Figure 3:**
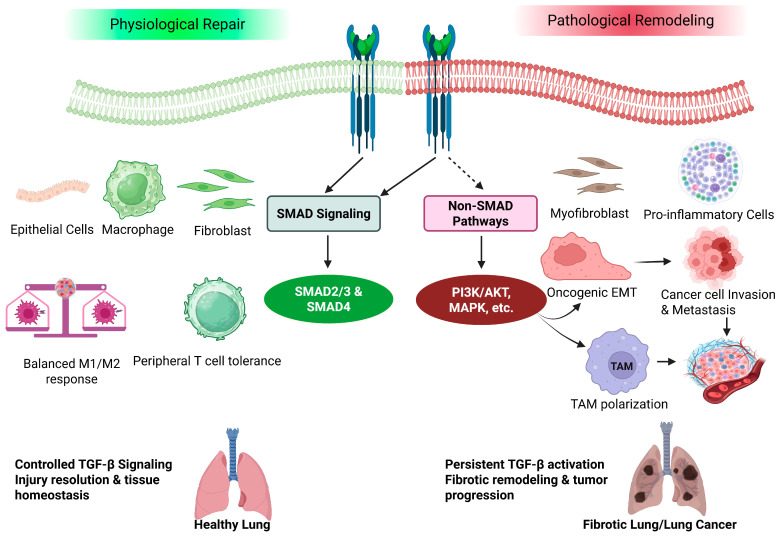
Context-dependent roles of TGF-β signaling in lung tissue repair, pulmonary fibrosis, and lung cancer progression. Under physiological conditions, transient and spatially restricted activation of TGF-β signaling following acute epithelial injury promotes controlled epithelial regeneration, resolution of inflammation, and reversible fibroblast activation. Negative feedback mechanisms, including inhibitory SMADs, ensure timely signal termination, thereby restoring tissue homeostasis and maintaining normal lung architecture. In contrast, repetitive injury or dysregulated microenvironmental cues lead to sustained and amplified TGF-β activation, characterized by persistent SMAD2/3 phosphorylation and engagement of non-canonical pathways (e.g., PI3K/AKT and MAPK signaling). Chronic signaling drives fibroblast-to-myofibroblast transition, excessive extracellular matrix (ECM) deposition, epithelial plasticity, and immune dysregulation, resulting in progressive pulmonary fibrosis. In the oncogenic context, sustained TGF-β activity further promotes epithelial–mesenchymal transition (EMT), tumor cell invasion, immune suppression, and metastatic progression, establishing a pro-tumorigenic microenvironment. This figure was created in BioRender.

Moreover, the duration and intensity of TGF-β signaling are increasingly recognized as critical determinants distinguishing physiological tissue repair from pathological fibrosis. During normal wound healing, TGF-β activation is typically transient and reversible, diminishing as inflammatory stimuli resolve. In contrast, in IPF persistent stimuli—including recurrent epithelial injury, increased mechanical tension, and feedback from a stiffened ECM—establish a positive feedback loop that sustains the long-term activation of both SMAD and non-SMAD pathways. This self-reinforcing signaling state ultimately drives irreversible tissue remodeling and progressive loss of lung function (Figure 3) [13,76,77].

Collectively, aberrant TGF-β signaling in IPF is characterized by sustained pathway activation and extensive crosstalk amplification, highlighting downstream signaling dynamics as key therapeutic targets. These functional insights are further supported by genetically engineered mouse models. For example, mice harboring surfactant protein C (SP-C) mutations develop spontaneous alveolar epithelial injury and TGF-β-dependent fibrosis, confirming a causal role of TGF-β in fibrotic progression [78]. In addition, SMAD3 knockout mice exhibit reduced myofibroblast differentiation and attenuated ECM deposition following bleomycin challenge, underscoring the critical mediatory function of SMAD3 in pulmonary fibrosis [79].

### 6.2. TGF-β Pathway Activation and Lung Cancer Progression

Under physiological conditions, TGF-β signaling plays a critical role in maintaining lung tissue homeostasis by regulating epithelial cell differentiation, controlling immune cell activation, and limiting excessive inflammatory responses [80]. In a normal lung, basal TGF-β activity contributes to epithelial integrity and immune tolerance by restraining aberrant proliferation of epithelial cells and modulating cytotoxic T-cell and macrophage functions [80,81]. Disruption of this tightly regulated signaling balance, particularly sustained or excessive pathway activation, shifts TGF-β signaling from a homeostatic regulator toward a driver of pathological remodeling and tumor progression [82].

Importantly, TGF-β signaling exhibits a well-established tumor suppressor role during early stages of epithelial carcinogenesis. In premalignant or early-stage epithelial cells, TGF-β induces cytostatic responses by promoting cell-cycle arrest through upregulation of CDK inhibitors such as p15^INK4b^ and p21^CIP1^, and by repressing MYC transcription, thereby limiting uncontrolled proliferation [83,84]. Genetic studies have demonstrated that disruption of core TGF-β pathway components—including TGFBR2 or SMAD4—facilitates tumor initiation and progression, underscoring the physiological tumor-suppressive function of intact TGF-β signaling in early carcinogenesis [85,86].

In NSCLC, TGF-β signaling exhibits pronounced context dependency. Numerous studies have demonstrated that, during tumor progression, the activation of TGF-β signaling is closely associated with the EMT, enhanced invasiveness, and increased metastatic potential [87]. Even in tumor specimens lacking detectable mutations in SMAD or TGFBR genes, TGF-β can promote tumorigenesis through the induction of EMT-associated transcription factors such as SNAIL and ZEB1 [88,89]. This stage-dependent shift reflects reprogramming of downstream signaling outputs rather than a binary change in pathway activity. Mechanistically, oncogenic cooperation (e.g., RAS activation), alterations in SMAD co-factors, and engagement of non-canonical signaling branches can attenuate cytostatic responses while preserving or amplifying pro-invasive and immunosuppressive functions [90,91]. Additionally, within the lung tumor microenvironment, TGF-β suppresses CD8^+^ T-cell activity and promotes immunosuppressive states, thereby facilitating immune evasion. Similarly, these immunomodulatory effects arise predominantly from signaling activation rather than structural genetic alterations [92].

Upon ligand–receptor engagement, TGF-β induces phosphorylation and the nuclear translocation of SMAD2/3, leading to the transcriptional regulation of downstream target genes. In lung cancer, this canonical pathway primarily governs the EMT, tumor invasion, metastatic competence, and immune modulation. The experimental inhibition of SMAD3 activity has been shown to reduce EMT phenotypes and cellular invasiveness, underscoring the canonical SMAD pathway as a key driver of lung cancer progression [93,94].

Beyond canonical SMAD signaling, TGF-β also activates non-SMAD pathways such as PI3K/Akt, MAPK/ERK, and JNK, which have been implicated in both lung fibrosis and lung cancer progression. In human lung fibroblasts, TGF-β1 stimulates ERK1/2 phosphorylation, leading to increased α-SMA and collagen production, and ERK inhibition attenuates these effects, revealing a MAPK-dependent profibrotic mechanism [95]. In NSCLC cells, TGF-β1 increases phosphorylation of AKT and ERK1/2 and induces EMT marker changes; inhibition of PI3K or MEK1/2 reduces EMT, demonstrating a direct role of these non-SMAD pathways in TGF-β-mediated EMT and invasiveness [96]. Moreover, in primary human lung fibroblasts TGF-β1 activates PI3K/JNK/Akt signaling, and blockade of these kinases significantly reduces profibrotic gene expression, further confirming their involvement in non-canonical TGF-β signaling [73]. Additional studies indicate that modulation of PI3K/Akt signaling can influence β-catenin activity and EMT phenotypes downstream of TGF-β1 in lung cancer models [97].

Overall, TGF-β signaling in lung cancer integrates canonical SMAD-mediated transcriptional programs with non-canonical survival and adaptive pathways, collectively promoting invasion, immune escape, and metabolic plasticity. This signaling-level dysregulation represents a set of actionable therapeutic targets. Selective inhibition of SMAD-dependent or SMAD-independent pathways may effectively interfere with lung cancer progression (Figure 3) [98,99]. Complementary evidence from genetically engineered mouse models further supports the causal role of TGF-β in lung tumor progression. Conditional knockout of *TGFBR1* or *TGFBR2* in lung epithelial cells modifies EMT induction and tumor development, while inducible overexpression of TGF-β in lung epithelial cells recapitulates fibrosis-to-tumorigenesis transitions, providing mechanistic and translational validation for the signaling-level observations described above [100,101].

## 7. A TGF-β–Driven Shared Pathological Axis Linking IPF and Lung Cancer: A Temporal Model from Acute Repair to Chronic Carcinogenesis

In this section, we propose a temporally stratified model illustrating how TGF-β signaling shifts from physiological repair to fibrosis and tumorigenesis (Figure 1).

### 7.1. Acute Lung Injury: TGF-β as a Physiological Regulator of Tissue Repair

Following an acute lung injury—such as an infection, an inhalational insult, or transient inflammation—TGF-β1 is transiently and locally activated. At this stage, TGF-β signaling is characterized by a low intensity and short duration, serving primarily to facilitate tissue repair, promote inflammation resolution, and maintain alveolar epithelial homeostasis while keeping the fibroblast activation in a reversible state.

During this phase, TGF-β signaling supports alveolar epithelial cell proliferation and differentiation, enabling the rapid restoration of the injured epithelial barrier. Simultaneously, inhibitory SMAD (e.g., SMAD7) provide negative feedback to prevent excessive or prolonged pathway activation. Numerous reviews have highlighted that TGF-β plays essential roles in lung remodeling, fibrosis, and tumorigenesis; however, its biological effects are highly context- and time-dependent [4].

### 7.2. Chronic Injury and IPF Development: Sustained Amplification of TGF-β Signaling

When a lung injury becomes repetitive or inadequately resolved, TGF-β1 activation shifts from a transient and tightly controlled response to a persistent, high-intensity signaling state. Recent mechanistic research revealed that non-canonical WNT5A/JNK/ROCK signaling promotes activation of latent TGF-β, driving fibroblast–myofibroblast transition and persistent fibrosis even without gene mutations, highlighting latent TGF-β activation as a key mechanism in IPF progression [102].

Through canonical SMAD2/3 signaling, TGF-β drives the fibroblast-to-myofibroblast transition (FMT) and upregulates ECM genes such as α-SMA and collagens. These mechanisms have been robustly demonstrated in bleomycin-induced and other experimental pulmonary fibrosis models [66].

With continued signaling, fibroblasts become progressively fixed as myofibroblasts, producing excessive collagen and fibronectin, while epithelial cells adopt partial EMT phenotypes [103]. Concurrently, immune responses shift from acute inflammation toward chronic dysregulation and immunosuppressive feedback loops. Collectively, these processes establish the characteristic fibrotic microenvironment of IPF and create fertile ground for subsequent tumorigenesis [104].

Importantly, multiple mechanistic studies indicate that non-SMAD pathways—including PI3K/AKT, MAPK/JNK, and Rho GTPase signaling—are co-activated during this stage and engage in extensive crosstalk with SMAD2/3 signaling, thereby reinforcing and stabilizing the fibrotic response [76]. Pharmacological inhibition of both SMAD and non-SMAD arms (PI3K/Akt, NF-κB) reduces fibrosis in experimental models, underscoring the functional significance of these synergistic pathways in driving the fibrotic program [66].

### 7.3. The Emergence of a Pro-Tumorigenic Microenvironment: The Pathological Continuum from IPF to Lung Cancer

In advanced disease stages, high-intensity and long-duration TGF-β signaling progressively endows the fibrotic microenvironment with pro-tumorigenic properties. These changes occur across multiple biological levels.

At the epithelial level, chronic TGF-β activation promotes EMT programs, increases cellular motility, and enhances genomic instability, thereby providing a mechanistic foundation for a premalignant transformation [105]. A TGF-β1–induced EMT is a well-recognized feature of NSCLC and is mediated through both SMAD2/3-dependent and non-SMAD pathways [97].

At the microenvironmental and immune levels, TGF-β not only drives fibroblast activation and ECM remodeling but also exerts profound immunoregulatory effects that collectively establish an immunosuppressive niche permissive for tumor initiation and progression. Sustained TGF-β signaling has been shown to impair innate immune surveillance by downregulating NKG2D ligands on lung epithelial and cancer cells, thereby attenuating NK cell–mediated cytotoxicity [106]. Moreover, TGF-β actively promotes the expansion and functional maturation of immunosuppressive myeloid-derived suppressor cells (MDSCs), which inhibit T cell proliferation through arginase-1-dependent mechanisms [107].

Importantly, chronic TGF-β signaling directly suppresses cytotoxic CD8^+^ T cell function by inhibiting effector differentiation, reducing granzyme B and IFN-γ production, and limiting clonal expansion within fibrotic and tumor-associated microenvironments. Experimental models have demonstrated that blockade of TGF-β signaling restores CD8^+^ T cell–mediated antitumor immunity and enhances responsiveness to immune checkpoint inhibition, underscoring a causal role of TGF-β in CD8^+^ T cell exclusion and dysfunction [108].

In addition, chronic TGF-β exposure not only induces immune checkpoint expression, including programmed cell death protein 1 (PD-1) on macrophages and programmed death-ligand 1 (PD-L1) within the tumor microenvironment [109], but also actively reprograms macrophages toward an immunosuppressive tumor-associated macrophage (TAM) phenotype [81,110]. Through sustained signaling cascades, TGF-β drives M2-like TAM differentiation characterized by increased IL-10 and TGF-β production, concomitant with elevated PD-1/PD-L1 expression. Together, these effects reinforce immune evasion by suppressing cytotoxic T-cell activity and reshaping both innate and adaptive immune compartments, ultimately shifting the balance from immune surveillance toward immune tolerance (Figure 1 and Figure 3).

Supporting this pathological continuity, cross-disease model analyses have identified shared downstream signaling networks. For example, osteopontin (SPP1) has been shown to promote both IPF and NSCLC progression via the PI3K/Akt/mTOR axis, underscoring the cumulative impact of multi-pathway TGF-β signaling crosstalk in disease evolution [111]. Clinically, large cohort and registry-based studies consistently demonstrate that patients with IPF exhibit a markedly elevated risk of developing lung cancer compared with the general population, with reported incidence rates approximately 3–7-fold higher. Notably, tumors frequently arise within peripheral fibrotic or honeycombed regions, supporting the concept that chronic fibrotic remodeling creates a field of carcinogenic susceptibility rather than representing a coincidental coexistence of two independent diseases [112,113].

### 7.4. Integrated Model Summary: TGF-β as a Temporal Master Regulator

Taken together, the proposed shared IPF–lung cancer pathological axis emphasizes that the disease trajectory is dictated not by the duration and regulatory balance of TGF-β signaling. Transient and tightly regulated TGF-β activation supports physiological tissue repair, whereas prolonged, multi-pathway-amplified signaling inexorably drives the lung toward irreversible fibrosis and carcinogenesis [76].

## 8. Therapeutic Strategies Targeting the TGF-β Pathway in Clinical Settings: The Current Status and Challenges

TGF-β plays a central role in the pathogenesis of IPF and lung cancer by driving fibrosis, EMT, immunosuppressive microenvironment remodeling, and tumor progression. Based on this pathogenic framework, therapeutic strategies targeting TGF-β signaling and its associated pathways have entered clinical development, demonstrating notable translational relevance and increasing clinical research momentum in both IPF and lung cancer (Table 4).

### 8.1. Direct Neutralization of TGF-β Ligands or Blockade of TGF-β Receptors

Fresolimumab (GC1008) is a humanized monoclonal antibody capable of neutralizing all three TGF-β isoforms (TGF-β1, β2, and β3) and has been evaluated in early-phase clinical trials for IPF and other fibrotic disorders. Although these studies demonstrated acceptable safety and tolerability, a definitive improvement in pulmonary function among IPF patients has not been firmly established, and no advanced-phase success has yet been reported. Further investigations are required to identify optimal patient subgroups and dosing regimens [114].

An alternative approach involves RNA-based therapeutic strategies. TRK-250, an antisense oligonucleotide that targets TGF-β mRNA, aims to directly reduce TGF-β production at the transcriptional and translational levels and is currently in early clinical development [115].

With respect to receptor blockades, galunisertib (LY2157299) is a small-molecule inhibitor of ALK5 (TGF-β receptor I kinase) that suppresses downstream SMAD2/3 phosphorylation and formation of the SMAD2/3–SMAD4 transcriptional complex [114,117,120,121]. By attenuating nuclear translocation and transcription of TGF-β–responsive profibrotic and pro-tumorigenic genes, including those involved in EMT, ECM production, and immune suppression, galunisertib aims to reduce sustained pathological signaling while preserving basal physiological TGF-β activity, potentially improving tolerability compared with ligand-neutralizing strategies. Clinical evaluation in IPF is ongoing, and studies in selected tumors have demonstrated favorable safety and early biomarker modulation.

In addition, bifunctional or bispecific antibodies combining TGF-β inhibition with immune checkpoint blockades (e.g., PD-1 or PD-L1 targeting) have been developed to simultaneously enhance antitumor immunity and attenuate fibrosis. Preclinical and early clinical studies suggest potential synergistic benefits; however, robust clinical evidence is still required to validate these approaches [122].

### 8.2. Inhibition of Upstream Activation and Reduction in TGF-β Activation

The activation of latent TGF-β often requires integrins such as αvβ6 and αvβ1, making these molecules attractive therapeutic targets. PLN-74809, a dual αvβ6/αvβ1 integrin inhibitor, reduces TGF-β activation, attenuates SMAD3 phosphorylation, and suppresses fibrotic gene expression. This compound is currently under Phase II clinical evaluation for IPF [115,116].

Other anti-integrin strategies, such as BG00011, have also been assessed in early-phase trials regarding safety and TGF-β pathway modulation; however, available data remain limited and inconclusive [123].

### 8.3. Indirect Blockade or Modulation of Downstream Pathways

Several approved anti-fibrotic agents exert indirect inhibitory effects on the TGF-β pathway.

(i) Pirfenidone, an oral anti-fibrotic drug approved by the U.S. FDA and European regulatory agencies for IPF, exhibits pleiotropic mechanisms, including the suppression of TGF-β1 expression, inhibition of Smad3 signaling, reduction in fibroblast activation, and attenuation of ECM deposition. Clinical studies have demonstrated its ability to slow lung function declines and reduce mortality risks [72].
(ii) Nintedanib, a multitarget receptor tyrosine kinase inhibitor (targeting platelet-derived growth factor receptor, vascular endothelial growth factor receptor, fibroblast growth factor receptor, etc.), has demonstrated efficacy in Phase III clinical trials by reducing the rate of the lung function decline and acute exacerbations in IPF. Although not a direct TGF-β inhibitor, it may exert modulatory effects on TGF-β-related pathways [72].

Another indirect strategy involves targeting downstream mediators such as CTGF. Pamrevlumab (FG-3019), a monoclonal antibody against CTGF, has demonstrated potential improvements in lung function and quality of life measures in chronic IPF studies and is currently being evaluated in large-scale Phase III trials [115].

### 8.4. TGF-β-Targeted Strategies in Lung Cancer

In lung cancer clinical development, TGF-β-targeted strategies have primarily focused on the following: (i) Fusion proteins or bispecific antibodies, such as bintrafusp alfa, which combines a PD-L1 blockade with a TGF-β “trap” mechanism and has been evaluated for its efficacy and safety in patients with advanced NSCLC during early- and mid-phase clinical trials [118]. (ii) Small-molecule TGF-βR1 kinase inhibitors, including vactosertib (LY3200882), which have demonstrated acceptable safety profiles and preliminary evidence of antitumor biomarker modulation in early-phase studies of solid tumors, including NSCLC [119]. (iii) Although some trials involving TGF-β-neutralizing antibodies (e.g., fresolimumab, NIS793) in solid tumors, including lung cancer, have demonstrated limited clinical benefits or have been discontinued, these experiences highlight the need for further optimization and rational combination strategies [118].

Overall, direct TGF-β inhibition as a monotherapy has demonstrated limited efficacy in lung cancer. Consequently, current clinical development has increasingly shifted toward combination approaches with immunotherapy, chemotherapy, or radiotherapy to overcome immunosuppressive tumor microenvironments and enhance therapeutic responses. Given the multifunctional role of TGF-β in normal physiological regulation, indiscriminate inhibition may disrupt tissue repair and immune homeostasis, leading to adverse effects or limited tolerability. Therefore, distinguishing pathological activation from physiological TGF-β signaling and precisely targeting specific cellular contexts (e.g., fibroblasts versus tumor microenvironment) remain critical challenges. Multitarget combination therapies, bifunctional antibodies, and patient stratification strategies are emerging as key directions in contemporary clinical trial designs [98].

Several combination strategies have demonstrated encouraging signals in early-phase trials. For example, bintrafusp alfa, a bifunctional fusion protein combining PD-L1 blockade with a TGF-β trap, has shown durable responses in subsets of advanced NSCLC patients in Phase I studies [124]. Similarly, vactosertib combined with immune checkpoint inhibitors has demonstrated enhanced antitumor immune activation in solid tumors [125]. In fibrotic disease, integrin inhibitors targeting upstream TGF-β activation are being explored alongside standard anti-fibrotic agents to enhance pathway modulation. These emerging data support the concept that multitarget approaches may overcome the limitations observed with TGF-β–directed monotherapy.

### 8.5. Temporal and Context-Dependent Targeting of TGF-β Signaling: Implications for Therapeutic Intervention

Accumulating experimental evidence indicates that persistent activation of TGF-β signaling, rather than transient or physiological activation, acts as a causal driver of pathological progression in both fibrosis and cancer. For example, Wu et al. demonstrated that a TGF-β1–RCN3–TGFBR1 feed-forward loop maintains prolonged pathway activation, thereby exacerbating fibrotic remodeling in the lung [23]. Similarly, genetic disruption of αv integrin–mediated TGF-β1 activation markedly attenuates radiation-induced pulmonary fibrosis, underscoring that the level and duration of bioactive TGF-β critically determine fibrotic severity [126]. Collectively, these findings establish sustained TGF-β signaling as a mechanistic determinant of disease progression rather than a mere bystander response.

Preclinical intervention studies further support the concept that therapeutic modulation of TGF-β signaling is most effective when applied during active or progressive stages of disease. In bleomycin-induced pulmonary fibrosis models, Wang et al. showed that overexpression of the long non-coding RNA growth arrest-specific 5 suppresses TGF-β1–induced pericyte-to-myofibroblast transition, resulting in reduced fibrotic burden [127]. Pharmacological studies provide complementary evidence: Yao et al. demonstrated that sinomenine attenuates pulmonary fibrosis by downregulating both Smad-dependent and non-Smad (PI3K/Akt) TGF-β signaling pathways [66]. Importantly, these interventions target pathway activity and persistence, rather than irreversible genetic alterations, indicating that TGF-β–driven fibrosis remains therapeutically tractable within a defined temporal window.

In cancer, the therapeutic relevance of timing and context is particularly evident in the dual role of TGF-β signaling. While early-stage tumors may retain TGF-β–mediated growth suppression, advanced cancers often exploit sustained TGF-β activity to induce EMT, immune evasion, and treatment resistance. Recent work by Sun et al. demonstrated that selective inhibition of TGF-β–induced EMT restores chemotherapy sensitivity in lung squamous cell carcinoma models, without globally abrogating TGF-β signaling [128]. These findings highlight that context-specific and temporally controlled targeting of pathological TGF-β outputs—rather than complete pathway blockade—may provide superior therapeutic benefit, particularly in advanced or therapy-resistant disease states.

Together, these studies support a unifying model in which the duration, intensity, and cellular context of TGF-β signaling determine its pathological versus protective roles. This framework provides a strong rationale for time- and stage-dependent therapeutic strategies, emphasizing selective modulation of sustained or maladaptive TGF-β signaling as a promising avenue for clinical intervention in both fibrotic diseases and cancer.

## 9. Conclusions and Future Perspectives: Integrating the Clinical Translational Value of TGF-β in Pulmonary Fibrosis and Lung Cancer

This review systematically explores the central role of TGF-β signaling in pulmonary fibrosis and lung cancer, integrating molecular mechanisms, pathological dynamics, and emerging clinical therapeutic strategies. TGF-β is a multifunctional cytokine that contributes to tissue repair, immune regulation, and growth suppression in a normal lung. However, under pathological conditions, the dysregulated activation of TGF-β signaling promotes interstitial remodeling, EMT, immune evasion, and tumor progression [4].

In IPF, TGF-β serves as a key driver of fibroblast-to-myofibroblast differentiation and fibrosis progression by inducing excessive extracellular matrix deposition and tissue stiffening. Experimental and histopathological studies have consistently shown that elevated TGF-β activity correlates with a declining lung capacity, impaired gas exchange, and increased interstitial rigidity. Recent animal models and organoid studies further indicate that injured alveolar type II epithelial cells can sustain fibrosis through autocrine TGF-β activation loops [129].

Within the clinical–pathological context of lung cancer and IPF comorbidity, TGF-β not only amplifies fibrotic mechanisms but also plays a pivotal role in shaping the tumor microenvironment through its potent EMT-inducing and immunosuppressive functions. Pathological TGF-β activation promotes CAF expansion, extracellular matrix remodeling, and the suppression of effector immune cell activity, collectively creating a pro-tumorigenic niche [67]. Elevated TGF-β expression in lung cancer patients has also been associated with unfavorable clinical outcomes, including reduced overall survival and accelerated disease progression [9].

From a therapeutic perspective, strategies targeting TGF-β signaling are evolving from purely molecular interventions toward clinically applicable approaches. In IPF, anti-fibrotic agents such as pirfenidone and nintedanib, although not specific TGF-β inhibitors, attenuate TGF-β-associated downstream effects and have been clinically validated to slow functional decline and improve patient outcomes. More selective approaches, including integrin inhibitors and receptor kinase inhibitors, have demonstrated promising safety and efficacy signals in clinical trials (see the Table 4 in the Section 8). In lung cancer, combination strategies integrating TGF-β inhibition with immunotherapy or chemotherapy are emerging as a major focus, particularly in non-small cell lung cancer, where overcoming immunosuppressive microenvironments is critical for therapeutic success [118].

The addition of chemotherapy may enhance TGF-β–targeted therapy by increasing tumor antigen release and promoting immunogenic cell death, while TGF-β–induced EMT inhibition can restore chemosensitivity [98,130]. Safety considerations remain critical, as combined TGF-β and immune checkpoint inhibition may increase immune-related adverse events (e.g., dermatitis, colitis, pneumonitis), and overlapping toxicities with chemotherapy (myelosuppression, fatigue, hepatotoxicity) may limit tolerability; systemic TGF-β suppression may also impair tissue repair and cardiovascular homeostasis [131].

Ongoing trials are evaluating dual-pathway inhibition in NSCLC, including bintrafusp alfa (phase I NCT02517398 [124]; phase III NCT03631706 [132]), bintrafusp alfa with chemoradiotherapy (phase II NCT03840902) [133], and NIS793 combined with spartalizumab (NCT02947165) [134]. These studies aim to enhance response durability and overcome resistance relative to monotherapy.

Despite these advances, significant challenges remain in translating TGF-β-targeted therapies into routine clinical practice. The dual physiological and pathological roles of TGF-β necessitate precision targeting to avoid the disruption of normal tissue repair and immune homeostasis. Moreover, patient heterogeneity and disease stage variability limit the efficacy of single-target approaches, underscoring the need for robust biomarkers and refined patient stratification strategies to identify individuals most likely to benefit from TGF-β–directed interventions.

Future research directions include (1) precision biomarker development and patient stratification to identify subpopulations with pronounced pathological TGF-β activation; (2) selective cross-pathway inhibition, combined targeting of SMAD-dependent and complementary pathways such as PI3K/AKT or MAPK; and (3) a rational integration with immunotherapy, particularly immune checkpoint inhibitors, to enhance antitumor responses and overcome immune suppression.

It should be acknowledged that the continuum model proposed in this review, while biologically plausible and supported by cross-sectional molecular and experimental evidence, remains partially conceptual. The temporal and stage-dependent transitions linking acute repair, chronic fibrosis, and tumorigenesis have not yet been fully validated in longitudinal clinical studies. Future prospective investigations integrating multi-omics profiling and long-term patient follow-up will be essential to substantiate this framework and refine its translational applicability.

While its clinical translation poses substantial challenges, integrated, context-specific, and combinatorial therapeutic strategies hold considerable promise for transforming TGF-β-targeted interventions into effective and broadly applicable clinical treatments.

## Figures and Tables

**Table 1:** Literature-based summary of genomic-scale comparison of TGF-β pathway gene alterations in lung cancer and IPF.

Gene	Reported Variant Type in TCGA/Lung Cancer	Presence in IPF GWAS Association	Remarks/Interpretation	Representative Source
*TGFB1*	Higher expression/deregulation in tumors; not frequently somatically mutated	GWAS do not show TGFB1 as a top susceptibility locus	TGF-β1 is often dysregulated in expression and signaling but is not a common driver mutation in TCGA NSCLC datasets	Pan-cancer TCGA analysis (alterations include expression and methylation) as reported in [51]
*TGFBR1*/*TGFBR2*	Rare somatic mutations across cancers; not frequent in lung cancer	IPF GWAS do not identify these as top loci	Receptor gene somatic mutation frequency low (TCGA) and not GWAS-associated in IPF	Pan-cancer genomics review [51]
*SMAD2*/*SMAD3*	Rare coding mutations; no high-frequency hotspots in NSCLC	Not identified in major IPF GWAS loci	Core signal transducers; impacted through pathway activity changes and epigenetic dysregulation, not a direct SNP risk in IPF	Pan-cancer TCGA analysis as reported in [51]
*SMAD4*	Reported SMAD4 mutations in NSCLC (~5% in some cohorts) correlated with poor prognosis	Not a top IPF risk locus	SMAD4 alterations occur in a minority of NSCLC cases and affect TGF-β signaling but are not identified as a common IPF GWAS locus	Clinical NSCLC mutation study [52]
Other *TGF-β* superfamily-related genes (e.g., *BMP5*, *ACVR2A*, *BMPR2*)	Some have mutations in pan-cancer analyses including TCGA; not lung-specific	No direct IPF GWAS signals	Found as variant hotspots in large pan-cancer analyses, indicating pathway involvement	TCGA pan-cancer analytic as reported in [51]
IPF GWAS Top-Risk Genes (e.g., MUC5B, TERT, DSP)	Not core TGF-β pathway genes	Identified as strong IPF susceptibility loci	Suggests IPF genetic risk is driven by non-TGF-β core signaling genes	IPF GWAS meta-analysis as reported in [53]

TCGA, The Cancer Genome Atlas; GWAS, genome-wide association studies; NSCLC, non-small cell lung cancer; and IPF, idiopathic pulmonary fibrosis. All entries are summarized from previously published TCGA- and GWAS-based studies.

**Table 2:** TGF-β pathway gene variants and lung fibrosis/lung cancer—gene-level evidence.

Gene	Type of Variation	Disease Context	Functional Effect/Mechanism	Representative Evidence
*TGFB1*	SNP: rs1800469 (C-509T)/rs1982073 (L10P)	Lung cancer susceptibility (population studies)	These promoter/peptide variants alter expression/levels of TGF-β1 and may modulate risk of NSCLC (some subgroups).	Meta-analysis: *TGF-β1* polymorphisms and lung cancer risk (rs509/rs1982073) show association in subgroup analyses [56]
*TGFBR2*	Promoter polymorphism (studied, low MAF)	Lung adenocarcinoma (case–control)	Promoter variants (e.g., G875A) investigated; may change expression but no strong association in studied populations.	*TGF-β2*/*TGFBR2* promoter SNP study in lung adenocarcinoma [58]
*SMAD3*	SNP: rs12102171 and other SNPs	NSCLC (survival prediction)	Certain SMAD3 variants associated with survival differences after therapy; suggests functional impact of variation on pathway efficiency.	*TGF-β* SNPs predict NSCLC overall survival [59]
*SMAD4*	Promoter hypermethylation (epigenetic)	Lung cancer in IPF	Reduced SMAD4 expression via promoter methylation observed in lung cancer/IPF, reducing growth inhibitory response to TGF-β.	Reduced Smad4 expression and promoter methylation in lung cancer/IPF cohort [60]
*SMAD7*	DNA hypermethylation (epigenetic)	Lung adenocarcinoma metastasis	*SMAD7* hypermethylation (via DNMT3B recruitment) suppresses its inhibitory effect → signaling hyperactivation → metastasis potential ↑.	PHF14 enhances *SMAD7* DNA methylation, promoting TGF-β–driven metastasis [61]
Other pathway SNPs (e.g., *BMP2*, *SMAD9*)	SNPs in pathway genes	NSCLC (survival models)	Certain downstream/related gene variants show statistical association with clinical outcomes, supporting polygenic contribution.	NSCLC SNP prediction study (*BMP2*/*SMAD9*) [59]

SNPs, single nucleotide polymorphisms; NSCLC, non-small cell lung cancer; MAF, minor allele frequency; and IPF, idiopathic pulmonary fibrosis. Arrow symbols indicate directional biological relationships: “→” denotes a sequential or causal effect, and “↑” indicates increased activity or outcome.

**Table 3:** Functional activation of TGF-β pathway components in pulmonary fibrosis and lung cancer.

TGF-β Pathway Component	Mode of Activation	Disease Context	Major Biological Effects	Representative Evidence
TGFB1	Increased expression and enhanced receptor stabilization	IPF	Promotes myofibroblast differentiation and excessive ECM deposition	PKM2 enhances TGF-β1 signaling and promotes pulmonary fibrosis via PKM2–Smad7–TβR1 interaction [65]
TGFBR1	Increased receptor abundance and downstream signal initiation	IPF	Activates canonical TGF-β/SMAD signaling and cooperates with non-canonical pathways	RCN3 enhances both canonical and non-canonical TGF-β signaling by upregulating TGFBR1 in pulmonary fibrosis [23]
SMAD2/3	Sustained phosphorylation and nuclear translocation	IPF	Upregulates ECM-related genes (e.g., α-SMA, collagens) and drives fibroblast activation	TGF-β1-induced fibroblast activation and ECM production mediated via SMAD2/3 signaling [66]
PI3K/AKT	Non-canonical pathway activation	IPF/NSCLC	Promotes cell proliferation, survival, apoptosis resistance, and invasive behavior; contributes to fibrosis and tumor progression	IPF: PI3K–AKT–mTOR signaling mediates EMT and ECM accumulation; NSCLC: pathway activation associated with tumor invasion and progression [67]
MAPK (p38/JNK/ERK)	Non-canonical signaling crosstalk with TGF-β	IPF/NSCLC	Enhances fibroblast transdifferentiation, EMT, and pro-fibrotic gene expression	TGF-β1 augments downstream gene expression via p38 and JNK MAPK signaling and PI3K pathway crosstalk [68]

IPF, idiopathic pulmonary fibrosis; ECM, extracellular matrix; NSCLC, non-small cell lung cancer; and EMT, epithelial–mesenchymal transition.

**Table 4:** Clinical-stage therapeutic agents targeting the TGF-β pathway.

Molecular Target/Drug	Mechanism of Action	Disease/Indication	Clinical Stage/NCT	Key References
Pirfenidone	Inhibition of TGF-β1 expression and Smad3 signaling	IPF	Marketed/multiple RCTs completed	[72]
Nintedanib	Multitarget tyrosine kinase inhibitor	IPF	Marketed	[72]
Fresolimumab (GC1008)	Neutralizing monoclonal antibody against TGF-β1/2/3	IPF/other fibrotic diseases	Phase I/early clinical	[114]
TRK-250	Antisense oligonucleotide targeting TGF-β mRNA	IPF	Phase I	[115]
PLN-74809	Dual αvβ6/αvβ1 integrin inhibitor	IPF	Phase II	[115,116]
Galunisertib (LY2157299)	TGF-βR1 (ALK5) kinase inhibitor	IPF/fibrosis	Phase II/early clinical	[114,117]
Pamrevlumab (FG-3019)	Anti-CTGF monoclonal antibody (downstream of TGF-β)	IPF	Phase III	[115]
Bintrafusp alfa	PD-L1 blockade plus TGF-β trap	NSCLC/solid tumors	Phase I/II	[118]
Vactosertib (LY3200882)	Small-molecule TGF-βR1 inhibitor	NSCLC/solid tumors	Phase I/II	[119]

IPF, idiopathic pulmonary fibrosis; RCTs, randomized controlled trials; and NSCLC, non-small cell lung cancer.

## Data Availability

No new data were created or analyzed in this study.

## Abbreviations

The following abbreviations are used in this manuscript:

IPF	Idiopathic pulmonary fibrosis
ECM	Extracellular matrix
EMT	Epithelial–mesenchymal transition
TGF-β	Transforming growth factor-β
CAF	Cancer-associated fibroblasts
NSCLC	Non-small cell lung cancer
SMAD	Sma- and Mad-related proteins
PD-1	Programmed cell death protein 1
NKG2DL	NKG2D ligand
PDGF	Platelet-derived growth factor
CTGF	Connective tissue growth factor
FGFs	Fibroblast growth factors
LAP	Latency-associated peptide
LTBPs	latent TGF-β–binding proteins
TβRII	Transmembrane type II TGF-β receptor
TβRI	Transmembrane type I TGF-β receptor
SMURF1	Smad ubiquitination regulatory factor 1
SNPs	Single nucleotide polymorphisms
SCLC	Small cell lung cancer
EGFR	Epidermal Growth Factor Receptor
KRAS	Kirsten Rat Sarcoma Viral Oncogene Homolog
TP53	Tumor Protein 53
TCGA	The Cancer Genome Atlas
GWAS	Genome-wide association studies
BLM	Bleomycin
SP-C	Surfactant protein C
FMT	Fibroblast-to-myofibroblast transition
MDSCs	Myeloid-derived suppressor cells
PD-L1	Programmed death-ligand 1
TAM	Tumor-associated macrophage
